# Evidence for harm reduction in COPD smokers who switch to electronic cigarettes

**DOI:** 10.1186/s12931-016-0481-x

**Published:** 2016-12-16

**Authors:** Riccardo Polosa, Jaymin Bhagwanji Morjaria, Pasquale Caponnetto, Umberto Prosperini, Cristina Russo, Alfio Pennisi, Cosimo Marcello Bruno

**Affiliations:** 1 Dipartimento di Medicina Clinica e Sperimentale, University of Catania, Catania, Italy; 2 Centro per la Prevenzione e Cura del Tabagismo (CPCT), “Policlinico - V. Emanuele”, University of Catania, Catania, Italy; 3 Internal and Emergency Medicine, “Policlinico - V. Emanuele”, University of Catania, Catania, Italy; 4 Department of Respiratory Medicine, Royal Brompton & Harefield Hospital Foundation Trust, Harefield Hospital, Hill End Road, Harefield, UB9 6JH UK; 5 Ospedale “San Vincenzo” - ASP Messina, Taormina (ME), Italy; 6 MCAU ARNAS Garibaldi, Catania, Italy; 7 U.F. Malattie Apparato Respiratorio, Casa di Cura Musumeci-Gecas, Gravina di Catania, Italy

**Keywords:** Smoking cessation, Electronic cigarette, COPD, Tobacco harm reduction

## Abstract

**Background:**

Electronic cigarettes (ECs) are battery-operated devices designed to vaporise nicotine, which may help smokers quitting or reducing their tobacco consumption. There is a lack of data on the health effects of EC use among smokers with COPD and whether regular use results in improvement in subjective and objective COPD outcomes.

We investigated long-term changes in objective and subjective respiratory outcomes in smokers with a diagnosis of COPD who quit or reduced substantially their tobacco consumption by supplementing with or converting only to ECs use.

**Methods:**

We conducted a retrospective chart review of patients with COPD to identify those reporting regular daily use of ECs on at least two follow-up visits at 12- (F/up1) and 24-months (F/up2). Regularly smoking COPD patients were included as a reference group.

**Results:**

A marked reduction in cigarette consumption was observed in ECs users. A significant reduction in COPD exacerbations was reported in the COPD EC user group, their mean (±SD) decreasing from 2.3 (±1) at baseline to 1.8 (±1; *p* = 0.002) and 1.4 (±0.9; *p* < 0.001) at F/up1 and F/up2 respectively. A significant reduction in COPD exacerbations was also observed in ECs users who also smoked conventional cigarettes (i.e. ‘dual users’). COPD symptoms and ability to perform physical activities improved statistically in the EC group at both visits, with no change in the control group.

**Conclusions:**

These findings suggest that ECs use may aid smokers with COPD reduce their cigarette consumption or remain abstinent, which results in marked improvements in annual exacerbation rate as well as subjective and objective COPD outcomes.

## Background

Chronic obstructive pulmonary disease (COPD) is a progressive disease characterized by a persistent inflammatory and remodelling response of the airways causing respiratory symptoms, progressive decline besides in lung function, respiratory failure, cor pulmonale and death [1–7]. COPD is estimated to become the third leading cause of death in 2030 (www.who.int/whosis/whostat/2008/en/). As expected, COPD and the catastrophic complications of advanced stage disease impose a substantial economic burden on healthcare systems; in the US alone, direct costs of COPD have been estimated at $29.5 billion, with indirect costs of $20.4 billion [8]. Studies in the UK have estimated an annual direct cost of treatment per patient of £819 [9].

The distinctive inflammatory response of the airways in COPD is generally associated with tobacco smoking [2, 3], with about 15–20% of smokers developing a diagnosis of COPD [4]. Furthermore, COPD smokers or ex-smokers are at an increased risk for lung cancer [5], cardiovascular diseases [6, 7] and diabetes [10].

Smoking cessation is the only evidence based strategy known to improve the COPD prognosis [11, 12]. Smoking cessation reduces the rate of annual decline in pulmonary function, attenuates respiratory symptoms of cough and sputum, and improves health status [13–15]. Additionally, stopping smoking reduces the risk of developing and eventually dying from lung cancer, cardiovascular disease and other tobacco-related illnesses [16]. Therefore, it is important to recommend COPD patients who smoke to quit as early as possible.

Although FDA-approved smoking cessation drugs (i.e. nicotine replacement therapy, buproprion, and varenecline) in combination with counseling have been shown to promote abstinence in COPD patients who smoke, the relapse rate is very high compared to smokers in the general population [17]. Unsuccessful smoking cessation and relapses are more frequently reported in COPD patients [18, 19], mainly because of their higher pack-year history, greater degree of nicotine dependence, inferior motivation to quit, and increased risk for depressive symptoms [20]. Improved quit rates would be desirable in a population that generally responds poorly to smoking cessation efforts. Consequently, the need for novel and more efficient approaches to smoking cessation interventions is unquestionable.

Electronic cigarettes (ECs) are battery-operated devices designed to vaporise nicotine without burning tobacco. ECs are now regulated in the EU by the new Tobacco Products Directive (TPD) [21], which mandates that e-vapour products are only placed on the market if the nicotine dose and uptake is reported and toxicological risk assessment is carried out on aerosol emissions. Marketing of ECs is now legal in the United States, where the FDA recently finalized rules for the regulation of ECs as a tobacco product (www.who.int/whosis/whostat/2008/en/).

These consumer products share many similarities with smoking in the behavioural aspect of their use [22]. Users are predominantly smokers, who report using them long-term as an alternative to conventional cigarettes, reduce their consumption or quit smoking, alleviate tobacco withdrawal symptoms, and continue having a ‘smoking’ experience [23], but mitigated health risks [24, 25]. Data from clinical trials [26–28] and meta-analyses [29] have shown that ECs may help smokers quitting or reducing their tobacco consumption and their use is well tolerated. There is a lack of data on the health effects of EC use amongst smokers with COPD in the literature. In particular, the impact of inhaling aerosol emissions from ECs on routinely assessed objective and subjective respiratory outcomes in COPD patients is unknown. Here we report, a 24-month follow-up of respiratory outcomes in smokers with a diagnosis of COPD who quit or reduced substantially their tobacco consumption by switching to regular ECs use.

## Methods

This retrospective study was conducted at four Italian hospitals over the period September 2013 to December 2015 in the outpatient setting. The study was approved by the ethics review board of the coordinating center (“Policlinico-Vittorio Emanuele Hospitals”) and informed consent was obtained from each patient.

### Patient population

A review of the case notes of patients with COPD regularly followed up was conducted. Patients reporting regular daily use of ECs (and if at all conventional cigarettes) at least two follow-up visits over a 24-months period were eligible to for inclusion. A second group of age- and sex-matched COPD patients reporting to be regular smokers (and not using ECs) over the same observation period was selected from the four participating clinics as reference (control) group.

The diagnosis of COPD was made according to the Global initiative for Chronic Obstructive Lung Disease (GOLD) criteria [1]. In particular, selected patients had to have a ≥30 pack year smoking history and an obstructive post-bronchodilator spirometry ratio (i.e. <70%) documented in their notes. Smokers with COPD followed-up at these outpatient clinics were regularly asked about their smoking behaviour and given brief advice about quitting smoking. If interested in being assisted in a quit attempt they were referred to the smoking cessation clinic.

### Study design

A physicians from each of the participating centres reviewed the clinical notes of patients attending the clinics. COPD patient data was extracted from the clinic visit immediately preceding [baseline visit] the first of the two follow-ups visits [follow-up visit 1 and 2]. In brief, data from the three clinic visits were collected and analysed. Follow-up visits 1 (F/up1) and 2 (F/up2) were carried out at 12 (±1.5) and 24 (±2.5) months after baseline visits, respectively.

### Study outcomes assessed

The primary outcomes of interest were: a) reduction in cig/day consumption; and b) number of exacerbations in the previous 12 months at each of the visits and how they may have changed over the 24-month period in the EC group compared to the control group. Secondary outcomes of interest were changes from baseline to the final follow-up visit in: a) lung function; b) COPD Assessment Test (CAT) scores; and c) 6-min walk distance (6MWD). In addition, changes in the relative proportion of COPD GOLD stages throughout the 24-months observation period were reported for both study groups as well as the change in mean FEV1 from baseline to F/up2.

### Study assessments

At each routine outpatient clinic visit, patients were assessed using a standard approach consisting of review of smoking history, respiratory ailments and exacerbations, clinical examination, vital signs (blood pressure, heart rate, body weight), post-bronchodilator spirometry and GOLD staging [1], completion of the CAT (www.CATestonline.org) and re-evaluation of treatment adherence and efficacy. The CAT is a validated, short (8-item) and simple patient-completed questionnaire developed for use in routine clinical practice to assess health status of patients with COPD [30]. A change of 2 units is considered to be the minimal clinical importance difference [31]. If deemed appropriate and amenable, a 6MWD test was conducted to measure the overall ability to perform daily physical activities [32].

For the purposes of the study, severe exacerbations were defined as those requiring a course of antibiotics and/or oral corticosteroids via their primary care physician, attendance to the emergency department for nebulisation and/or hospital admission for their respiratory symptoms with the requirement of antibiotics and oral corticosteroids. Spirometry was conducted post-bronchodilator for measurement of forced expiratory volume in 1 s (FEV1) and forced vital capacity (FVC), and the expiratory ratio computed as percentage (%FEV1/FVC).

Patients’ data at the outpatient visits were extracted from their medical record and entered into an electronic spreadsheet for statistical computation.

## Analyses

Parametric data were expressed as mean (±standard deviation (SD)) while non-parametric data expressed as median (interquartile range (IQR)). We also delineated data for single (exclusive ECs use) and ‘*dual users*’ (i.e. ECs users who also smoke conventional cigarettes). Statistical comparisons of parameters were assessed using student’s *T*-test and Wilcoxon-signed rank test depending on whether the data was parametric or not, respectively. Similar statistical analyses were conducted on dual and single users within groups from baseline. Missing measurements were not included in the analyses. With the study involving repeated parameter measurements, analysis of repeated measures with Bonferroni correction was conducted for between groups over the study period. A two-tailed p value of less than 0.05 was considered to indicate statistical significance. All analyses were performed with the Statistical Package for Social Science (SPSS for windows version 18.0, Chicago, IL, USA).

## Results

### Patient characteristics

Data from a total of 48 COPD patients were included in the study. Patients had mild to very severe disease according to the GOLD criteria and were treated accordingly [1]. Twenty-four patients with COPD reporting regular daily EC use on two follow-up visits over the observation period of 24-months and twenty-four COPD matched controls were identified. Baseline demographic, COPD GOLD stage, objective and subjective parameter data on both study groups are summarised on Table 1. There were no significant differences in the measured parameters at baseline.

**Table 1:** Baseline demographics of the subjects on the study

	COPD Controls	COPD E-Cig users	Baseline *P*-value between groups
Age^c^	65.3 (±5.5)	66.9 (±6.7)	0.350
Sex	21 M, 3 F	20 M, 4 F	-
COPD GOLD stage			
Stage 1	3	2	-
Stage 2	5	6	-
Stage 3	11	10	-
Stage 4	5	6	-
post-BD FEV1^b^ (L)	1.47 (1.13, 1.72)	1.25 (0.94, 1.78)	0.298
post-BD FVC^b^ (L)	2.39 (2.1, 2.64)	2.37 (2, 2.65)	0.902
%FEV1/FVC^c^	56.2 (±10.3)	59.4 (±8.4)	0.244
Pack years of smoking^c^	51.7 (±9.9)	52.4 (±10.7)	0.365
Cig/day^c^	20.5 (±3.3)	21.8 (±4.4)	0.228
CAT score^b^	20.5 (17.8, 24.3)	21.5 (17.8, 25.3)	0.710
COPD Exacerbations^a, c^	2.1 (±1.1)	2.3 (±1)	0.440
Co-morbidities			
Respiratory failure	5	6	
CHF	3	3	
CHD	2	3	
Hypertension	9	8	
Diabetes	4	5	
OSAS	6	3	
Chronic Kidney Failure	1	0	
Liver cirrhosis	1	0	
Lung cancer	0	1	
Pulmonary hypertension	0	1	
GERD	3	2	
Degenerative joint disease	2	4	
Osteoporosis	2	1	
Depression/Anxiety	2	4	

*Abbreviations*: *COPD* Chronic obstructive pulmonary disease, *M* male, *F* female, *BD* bronchodilator, *L* litre, *FEV1* forced expiratory volume in 1 s, *FVC* forced vital capacity, *Cig* conventional cigarettes, *CAT* COPD assessment tool, *CHF* chronic heart failure, *CHD* coronary heart disease, *OSAS* obstructive sleep apnoea syndrome, *GERD* Gastroesophageal reflux disease

^a^ COPD exacerbations in past 12 months

^b^ Median (interquartile range); ^c^ Mean (± standard deviation)

### Changes in smoking behaviour and patterns of EC use

**Fig. 1:**
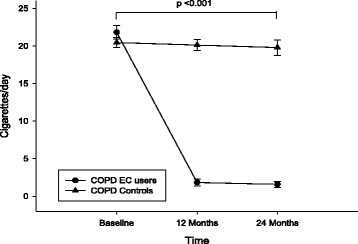
Changes in the number of cigarettes smoked in a day from baseline, at follow-up visit 1 (12 ± 1.5 months) and visit 2 (24 ± 2.5 months) separately for electronic cigarettes users (*closed circles*) and controls (*closed triangles*). All data expressed as mean and error bars are standard deviation of the mean. The p value is an overall comparison of both groups over the 24-month period

Patients’ cigarettes consumption at baseline and at follow-up visits are illustrated in Fig. 1. A substantial reduction in conventional cigarette consumption was observed in COPD EC users, their mean (±SD) cigarettes/day use decreasing from 21.8 (±4.4) at baseline to 1.8 (±2.2) at F/up1 and to 1.58 (±2.0) at F/up2, respectively (*p* < 0.001 for both visits) (Table 2). As expected, no significant change in conventional cigarette consumption was observed in the reference group.

**Table 2:** Comparison of controls and e-Cigarette users at baseline, 12-month and 24-month follow-up visits

	Baseline	12-Month Follow-up	Within group p value vs Baseline^Ω^	24-Month Follow-up	Within group *P* value vs Baseline^Ω^	Overall between group *p* value from Baseline^ƙ^
COPD Controls (*n* = 24)						
post-BD FEV1^a^ (L)	1.47 (1.13, 1.72)	1.43 (1.12, 172)	0.538	1.45 (1.17, 1.66)	0.657	0.223
post-BD FVC^a^ (L)	2.39 (2.1, 2.64)	2.35 (2.2, 2.74)	0.065	2.35 (2.19, 2.83)	0.141	0.977
%FEV1/FVC^b^	56.2 (±10.3)	55.9 (±10.1)	0.328	56.3 (±10.1)	0.277	0.033
Cig/day^b^	20.5 (±3.3)	20.1 (±3.7)	0.371	19.8 (±5)	0.296	<0.001
CAT score^a^	20.5 (17.8, 24.3)	20 (17.5, 24.3)	0.075	20 (15.8, 24)	0.361	0.001
COPD Exacerbations^b^	2.1 (±1.1)	2.2 (±1)	0.906	2.1 (±1.1)	0.819	0.005
6MWD^a, c^	267.3 (195, 351.5)	270 (210.3, 372)	0.056	270.5 (220.8, 373.9)	0.096	0.002
COPD EC users (*n* = 24)						
post-BD FEV1^a^ (L)	1.25 (0.94, 1.78)	1.23 (0.93, 1.73)	0.102	1.29 (0.92, 1.67)	0.153	
post-BD FVC^a^ (L)	2.37 (2, 2.65)	2.45 (1.92, 2.73)	0.081	2.46 (1.84, 2.86)	0.252	
%FEV1/FVC^b^	59.4 (±8.4)	58.3 (±8.6)	0.457	57.9 (±8.5)	0.483	
Cig/day^b^	21.8 (±4.4)	1.8 (±2.2)	<0.001	1.58 (±2)	<0.001	
CAT score^a^	21.5 (17.8, 25.3)	17.5 (15.8, 20.5)	<0.001	18 (15, 20)	<0.001	
COPD Exacerbations^b^	2.3 (±1)	1.8 (±1)	0.002	1.4 (±0.9)	<0.001	
6MWD^a, c^	266.5 (187.5, 313.5)	307 (219.5, 342)	0.002	327 (239.5, 359.5)	0.002	

*Abbreviations*: *COPD* Chronic obstructive pulmonary disease, *EC* e-Cigarette, *n* number, *BD* bronchodilator, *L* litre, *FEV1* forced expiratory volume in 1 s, *FVC* forced vital capacity, *Cig* conventional cigarettes, *CAT* COPD assessment tool, *6MWD* 6 min walk distance

^a^ Median (interquartile range); ^b^ Mean (± standard deviation)

^c^ 13 subjects in the COPD E-Cig user group and 14 in the COPD control group

^Ω^ Statistical analyses conducted using Mann Whitney *U* Test (as data non-parametric) except for Cig/day and COPD exacerbations which were analysed using student *T* test (parametric data)

^ƙ^ Statistical analyses conducted using repeated measures ANOVA with Bonferroni adjustment

Complete abstinence from tobacco smoking was observed in 13/24 (54.2%) of COPD EC users. Dual usage was reported by 11/24 (45.8%) COPD EC users. Nonetheless, a significant reduction in conventional cigarette consumption was also observed in dual users, with their mean (±SD) cigarettes/day use decreasing from 23.7 (±5.4) at baseline to 4 (±1.2) at F/up1 and to 3.5 (±1.3) at F/up2, respectively (*p* < 0.001 for both visits) (Table 3). More than 75% reduction from baseline in cigarette/day consumption was reported by all COPD EC dual users at both follow-up visits.

**Table 3:** Comparison of e-Cigarette and conventional cigarette users (dual users) vs e-Cigarette only users (single users) at 12- and 24-month follow-up visits

Parameter	Baseline	12-Month Follow-up	24-Month Follow-up
COPD EC users reducing cig use (dual users)	(*n* = 11)	(*n* = 11)	(*n* = 11)
Sex	10 M, 1 F	10 M, 1 F	11 M
% Smoking reduction compared to baseline	-	82.6 (±4.8)	85.1 (±4.7)
post-BD FEV1^a^ (L)	1.23 (0.94, 1.6)	1.20 (0.91, 1.70)	1.28 (0.92, 1.96)
post-BD FVC^a^ (L)	2.34 (2.04, 2.86)	2.35 (2.07, 2.89)	2.57 (2.22, 2.93)
%FEV/FVC^a^	50.9 (47, 61.2)	51.2 (46.9, 64)	51.8 (43.3, 65.7)
Cig/day^b^	23.7 (±5.4)	4 (±1.2)	3.5 (±1.3)
CAT score^a^	25 (19.5, 26.5)	20 (18, 22)	18 (15, 22)
COPD Exacerbations^b^	2.6 (±0.8)	2.3 (±0.8)	1.5 (±0.8)
COPD EC users ceasing cig use (single users)	(*n* = 13)	(*n* = 13)	(*n* = 13)
Sex	10 M, 3 F	10 M, 3 F	9 M, 3 F
Smoking reduction compared to baseline	-	-	-
post-BD FEV1^a^ (L)	1.32 (0.96, 1.76)	1.26 (0.94, 1.72)	1.3 (0.95, 1.63)
post-BD FVC^a^ (L)	2.57 (2.01, 2.65)	2.57 (1.92, 2.72)	2.44 (1.72, 2.82)
%FEV/FVC^a^	61.9 (50.8, 66.4)	61.5 (50.6, 65.6)	61.5 (50, 65.2)
Cig/day^b^	20.2 (±2.7)	-	-
CAT score^a^	20 (17, 24)	16 (14, 18)	17 (15, 20)
COPD Exacerbations^b^	2.07 (±1.0)	1.3 (±1)	1.4 (±1)

*Abbreviations*: *n* number, *COPD* Chronic obstructive pulmonary disease, *EC* e-Cigarette, *M* male, *F* female, *BD* bronchodilator, *L* litre, *FEV1* forced expiratory volume in 1 s, *FVC* forced vital capacity, *cig* conventional cigarettes, *CAT* COPD assessment tool

^a^Median (interquartile range); ^b^ Mean (± standard deviation)

### COPD exacerbations

**Fig. 2:**
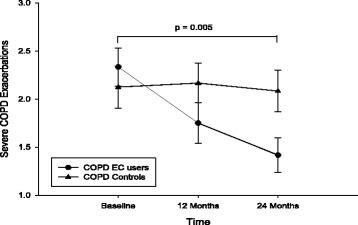
Changes in the number of COPD exacerbation from baseline, at follow-up visit 1 (12 ± 1.5 months) and visit 2 (24 ± 2.5 months) separately for electronic cigarettes users (*closed circles*) and controls (*closed triangles*). All data expressed as mean and error bars are standard deviation of the mean. The *p* value is an overall comparison of both groups over the 24-month period

There was a significant reduction in annual COPD exacerbations within the COPD EC user group, their mean (±SD) decreasing from 2.3 (±1) at baseline to 1.8 (±1; *p* = 0.002) at F/up1 and to 1.4 (±0.9; *p* < 0.001) at F/up2, while no significant change was observed in the control group (Table 2; Fig. 2). A between groups significant reduction (*p* = 0.005) in COPD exacerbations over the 24 months observation period was also noted (Table 2).

A significant reduction in COPD exacerbations was also observed in dual users, but only at 24 months; the number of exacerbation were reduced from 2.6 (±0.8) at baseline to 1.5 (±0.8; *p* = 0.002) at F/up2 (Table 3). In the single users there was marked reduction in exacerbations at F/up1 (*p* = 0.002) and F/up2 (*p* = 0.009) compared to baseline (Table 3). Of note, none of the patients included had a significant modification in COPD medications during the observation period.

### Lung function assessments and COPD staging

**Fig. 3:**
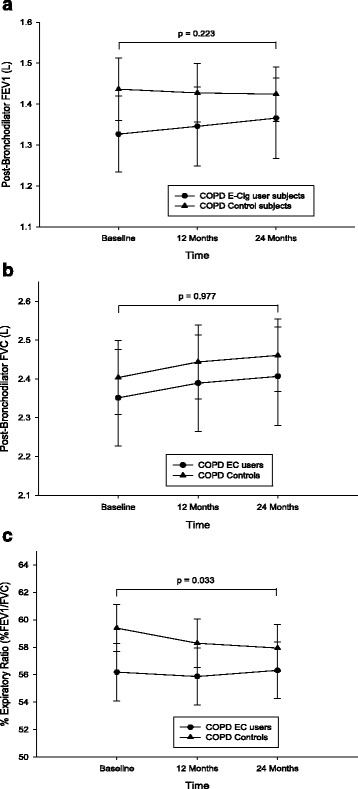
Changes in FEV1 (**a**), FVC (**b**), and %FEV1/FVC (**c**) from baseline, at follow-up visit 1 (12 ± 1.5 months) and visit 2 (24 ± 2.5 months) separately for electronic cigarettes users (*closed circles*) and controls (*closed triangles*). All data expressed as mean and error bars are standard deviation of the mean. The p value is an overall comparison of both groups over the 24-month period

Compared to baseline there were no significant differences in the post-bronchodilator FEV1, FVC and %FEV1/FVC between study groups (Table 2; Fig. 3a, b and c). There were no overall within group differences in spirometric assessments over the 24 month study period. Nonetheless, there was a significant difference (*p* = 0.037) in the rate of FEV1 decline at the 24-month follow-up visit in COPD ECs users (mean increase 39 mls) than in the control group (mean decrease 12 mls).

**Fig. 4:**
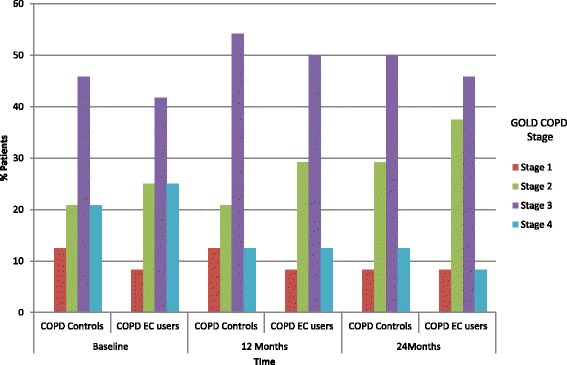
Bar chart representing COPD GOLD stage changes over the study period. NB: Twenty-four patients in each group

GOLD COPD staging variations are illustrated on Fig. 4. Over the 24-months observation period, we noted that a few COPD patients in the EC study group downstaged from GOLD Stage 4 to GOLD Stage 3 and 2. In contrast, the relative proportion of COPD GOLD stages for the reference group was virtually unchanged during the course of the study.

### CAT scores and 6MWD

**Fig. 5:**
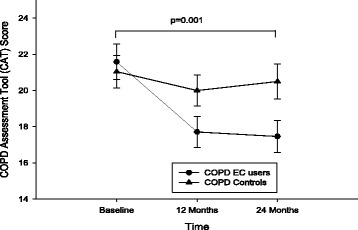
Changes in COPD Assessment Test (CAT) scores from baseline, at follow-up visit 1 (12 ± 1.5 months) and visit 2 (24 ± 2.5 months) separately for electronic cigarettes users (*closed circles*) and controls (*closed triangles*). All data expressed as mean and error bars are standard deviation of the mean. The p value is an overall comparison of both groups over the 24-month period

COPD symptoms, as assessed using the CAT, at both follow-up visits decreased statistically (both F/up1 and F/up2 *p* < 0.001) and clinically significantly (follow-up visit 1 and 2 reductions of 4 and 3.5 units, respectively) in the EC group, whereas there was little change in the control group (Table 2; Fig. 5). Similar overall between group significantly statistical improvements (*p* = 0.001) were noted in favour of the EC user group.

**Fig. 6:**
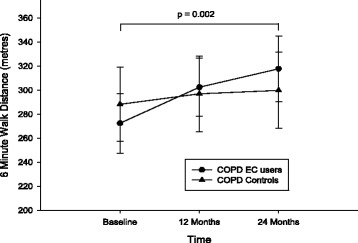
Changes in the 6-min walk distance (6MWD) test from baseline, at follow-up visit 1 (12 ± 1.5 months) and visit 2 (24 ± 2.5 months) separately for electronic cigarettes users (*closed circles*) and controls (*closed triangles*). All data expressed as mean and error bars are standard deviation of the mean. The p value is an overall comparison of both groups over the 24-month period

Results of 6MWD were only available in 13 patients of the EC user group and 14 of the control group (Table 2; Fig. 6). Over the 24 months observation period, the median 6MWD improved more than 60 m (*p* = 0.002) in the EC user group compared to just over a median of 3 m (*p* = 0.096) in the control group. Significant overall between group improvements were also noted in favour of the EC user group.

## Discussion

No formal efficacy assessment of EC use has been conducted in patients with COPD. Here, we show for the first time, albeit retrospectively, that COPD patients were able to quit or substantially reduced their tobacco consumption by switching to regular ECs use. In these patients we also document an improvement in several objective and subjective respiratory outcomes; in particular COPD exacerbations, annual decline in FEV1, CAT scores and 6MWD. Quality of life and attenuation of disease exacerbations was reported in a COPD patient switching ot vaping in a case series of three inveterate smokers [33]. A substantial reduction in conventional cigarette consumption was observed in COPD patients who switched to regular ECs use, with complete long-term abstinence from tobacco smoking being reported in over half of the COPD EC users. Dual usage was common (45.8%), though conventional cigarette consumption was substantially reduced, with all dual users smoking at least 75% less cigarettes compared to their baseline. Our observation of a 2-years abstinence rate of about 50% in a population, albeit small, that generally responds poorly to smoking cessation efforts is one of the highest ever reported in smoking cessation literature. The large magnitude of this effect in COPD may be explained by the fact that these products are known to replicate the smokers’ smoking experience and associated rituals, the great compensatory effect of EC at both physical and behavioral level is likely to explain the observed high success rates [22]. The same mechanism has been shown to drive key success rates among other vulnerable patients populations who switched to daily EC use, including asthma and schizophrenia [34–36].

Although smoking cessation is one of the few interventions shown to reduce all-cause mortality in patients with COPD [37], there is limited data showing the benefits of smoking cessation in reducing exacerbations. Our study is the first to consider the number of COPD exacerbations as an outcome in a smoking cessation study. We observed that in COPD patients who have switched to regular ECs use, there was a significant reduction in COPD exacerbations in exclusive EC users as well as dual users. These preliminary findings are in agreement with the reduced risk of COPD exacerbations of two large population studies [38, 39]. Godtfredson et al. reported that previous smokers had a 43% lower risk of hospitalization for COPD compared with current smokers [38]. Au et al. reported a 22% reduced risk of COPD exacerbations in ex-smokers compared with current smokers when adjusted for comorbidity, markers of COPD severity, and socioeconomic status [39]. By contrast, in the Lung Health Study [40] and in a 2.5-years follow up of 64 COPD patients by Kessler et al. [41] there was no significant difference in the risk of hospital admission between current smokers and ex-smokers. However, these studies were not consistent in considering influential confounders for the risk of COPD exacerbations such as duration of smoking abstinence, COPD severity, comorbidities, age, etc. In our investigation the two study groups were evenly mached for all these confounders. The marked attenuation in COPD exacerbations may be explained by the cessation/reduction in chronic exposure of the airways to cigarette smoke which is known to promote susceptibility to infection through a number of different mechanisms [42–44]; and switching to ECs is likely to lower the risk of respiratory infections and pneumonia [45]. Besides, regular vaping has been reported to favourably alter anti-microbial and -inflammatory activity in exhaled breath [46] besides the theoretical benefit of propylene glycol in its aerosol form being a potent bactericidal agent.

We did not observe any significant change in the post-bronchodilator FEV1, FVC and %FEV1/FVC within study groups. The lack of significant changes in standard spirometric indices after smoking cessation is not unusual in smokers with COPD [47, 48], which may be due to the pathophysiology associated with COPD [2, 3] especially in more advanced disease. Importantly, this is not the case in asthmatics in studies of comparable design [34, 35].

The effect of smoking on the progressive decline in lung function in COPD is well established [49] and attenuation in the annual rate of FEV1 decline has been generally reported in smoking cessation studies of COPD patients [13, 14]. In the current study, there was a significant reversal in the annual FEV1 decline at 24-month in COPD ECs users compared to COPD controls. Surprisingly, the improvement in the annual FEV1 decline was more so in the dual users than the single users; this was probably due to the higher proportion of less severe COPD GOLD stages in dual users.

Besides the observed reduction in exacerbation rates, amelioration of overall health status (as measured by CAT) and physical activity (measured by 6MWD) in the COPD patients who quit or reduced substantially their tobacco consumption by switching to regular ECs use are also novel and clinically relevant findings. Similar improvements in CAT scores and 6MWD have been shown in COPD patients undergoing intensive rehabilitation programs [31, 50]. The mechanism for these improved health outcomes following smoking cessation may be related to the substantial reduction in carbon monoxide (CO) (as well as in COHb levels) when giving up smoking [46] and the related time-dependent improvement in exercise tolerance upon smoking abstinence [51]. An internet based survey in COPD (*n* = 1190) and asthma (*n* = 1308) subjects has shown self-reported improved respiratory outcomes when switched to EC use of 75.7% and 65.4% respectively [23]. Surprisingly, with the use of ECs about a fifth of the all the subjects in the survey ceased to use any of their respiratory drugs and only around 1% of the asthmatics and COPD subjects had worsening respiratory symptoms.

There are some limitations in our study. Firstly, this is a relatively small retrospective study, hence the results cannot be generalized and must be interpreted with caution. Despite the small number of subjects we noted significant results in several crucial study endpoints. Standard concerns associated with retrospective studies (including variance in the quality of information recorded by medical professionals and difficulty in establishing a causal relationship) also need consideration. Nonetheless, a clear advantage conducting this type of study is the generation of hypotheses that can be tested prospectively under controlled conditions. Secondly, it is possible that patients in this study represent a self-selected sample, which may not be representative of all COPD smokers who tried ECs. Furthermore, assessment of smoking abstinence was self-reported and liable to recall bias. However, self-reported number of cigarettes smoked per day in studies of this type is not subjected to the kind of biases observed in clinical trials where there is the tendency to claim abstinence [52]. Moreover, similar beneficial effects were also reported in *dual users* (i.e., smoking reducers) and therefore objective measures of abstinence are unlikely to be of great importance. Additionally, the 6MWD was not conducted in all patients as it is not standard requirement and some patients declined.

## Conclusions

Regular ECs use may help smokers with COPD attenuate conventional cigarette consumption or remain abstinent, as well as improve subjective and objective COPD outcomes. The potential role of the e-vapor category for smoking cessation and/or harm reduction in COPD requires confirmation from larger prospective studies. Nonetheless, the notion that substitution of conventional cigarettes with ECs is unlikely to raise significant health concerns in COPD is generally reassuring and should be communicated to patients with COPD using or intending to use ECs. Moreover, given that smoking cessation is a behavioural transition not a biomedical cure for a disease, the approach should rest on the informed choice of COPD smokers and their view of what they think might work for them with the physician as an adviser rather than prescriber.

## Abbreviations

6MWD: 6 minute walk distance
CAT: COPD assessment tool
Cig/day: Conventianal cigarette per day
COPD: Chronic obstructive pulmonary disease
EC: Electronic cigarette
F/up: Follow-up
FDA: Food and Drug Agency
FEV1: Forced expiratory volume in 1 second
FVC: Forced vital capacity
GOLD: Global initiatve for Obstructive Lung Disease
IQR: Interquartile range
L: Litres
Mls: Millilitres
Post-BD: Post-bronchodilator
SD: Standard deviation

## References

[1] Global Initiative For Chronic Obstructive Lung Disease. Global Strategy for the Diagnosis, Management, and Prevention of Chronic Obstructive Pulmonary Disease (Updated 2008). published 2008. Url: http://goldcopd.org.

[2] MacNee W (2005). Pathogenesis of chronic obstructive pulmonary disease. Proc Am Thorac Soc.

[3] Morjaria JB, Malerba M, Polosa R (2010). Biologic and pharmacologic therapies in clinical development for the inflammatory response in COPD. Drug Discov Today.

[4] Fletcher C, Peto R (1977). The natural history of chronic airflow obstruction. Br Med J.

[5] Doll R, Hill AB (1950). Smoking and carcinoma of the lung; preliminary report. Br Med J.

[6] Falk JA, Kadiev S, Criner GJ, Scharf SM, Minai OA, Diaz P (2008). Cardiac disease in chronic obstructive pulmonary disease. Proc Am Thorac Soc.

[7] Finkelstein J, Cha E, Scharf SM (2009). Chronic obstructive pulmonary disease as an independent risk factor for cardiovascular morbidity. Int J Chron Obstruct Pulmon Dis.

[8] National Heart, Lung and Blood Institute (2009). Morbidity and Mortality: 2009 Chartbook of Cardiovascular, Lung and Blood Diseases.

[9] Chronic obstructive pulmonary disease. National clinical guideline on management of chronic obstructive pulmonary disease in adults in primary and secondary care. Thorax. 2004;59(Suppl 1):1–232. PMC1766028 15041752

[10] Chen W, Thomas J, Sadatsafavi M, FitzGerald JM (2015). Risk of cardiovascular comorbidity in patients with chronic obstructive pulmonary disease: a systematic review and meta-analysis. Lancet Respir Med.

[11] Fletcher C, Peto r (1978). Natural history of chronic respiratory tract obstruction. Bull Int Union Tuberc.

[12] Hersh CP, DeMeo DL, Al-Ansari E, Carey VJ, Reilly JJ, Ginns LC, Silverman EK (2004). Predictors of survival in severe, early onset COPD. Chest.

[13] Anthonisen NR, Connett JE, Kiley JP, Altose MD, Bailey WC, Buist AS, Conway WA, Enright PL, Kanner RE, O’Hara P (1994). Effects of smoking intervention and the use of an inhaled anticholinergic bronchodilator on the rate of decline of FEV1. The Lung Health Study. JAMA.

[14] Burchfiel CM, Marcus EB, Curb JD, Maclean CJ, Vollmer WM, Johnson LR, Fong KO, Rodriguez BL, Masaki KH, Buist AS (1995). Effects of smoking and smoking cessation on longitudinal decline in pulmonary function. Am J Respir Crit Care Med.

[15] Kanner RE, Connett JE, Williams DE, Buist AS (1999). Effects of randomized assignment to a smoking cessation intervention and changes in smoking habits on respiratory symptoms in smokers with early chronic obstructive pulmonary disease: the Lung Health Study. Am J Med.

[16] The Health Consequences of Smoking: 50 Years of Progress: A Report of the Sugeon General. In: Services. UDoHaH, editor. Atlanta: US Department of Health and Human Services, Centres for Disease Control and Prevention, National Centre for Chronic Disease Prevetnion and Health Promotion, Office on Smoking and Health; 2014.

[17] Tashkin DP (2015). Smoking Cessation in Chronic Obstructive Pulmonary Disease. Semin Respir Crit Care Med.

[18] Zhang MW, Ho RC, Cheung MW, Fu E, Mak A (2011). Prevalence of depressive symptoms in patients with chronic obstructive pulmonary disease: a systematic review, meta-analysis and meta-regression. Gen Hosp Psychiatry.

[19] van der Meer RM, Wagena EJ, Ostelo RW, Jacobs JE, van Schayck CP (2003). Smoking cessation for chronic obstructive pulmonary disease. Cochrane Database Syst Rev.

[20] Jimenez-Ruiz CA, Masa F, Miravitlles M, Gabriel R, Viejo JL, Villasante C, Sobradillo V (2001). Smoking characteristics: differences in attitudes and dependence between healthy smokers and smokers with COPD. Chest.

[21] DIRECTIVE 2014/40/EU OF THE EUROPEAN PARLIAMENT AND OF THE COUNCIL of 3 April 2014 on the approximation of the laws, regulations and administrative provisions of the Member States concerning the manufacture, presentation and sale of tobacco and related products and repealing Directive 2001/37/EC. J European Union. 2014. Accessed via: http://ec.europa.eu/health/tobacco/docs/dir_201440_en.pdf. Accessed 5 May 2016. 27660856

[22] Caponnetto P, Russo C, Bruno CM, Alamo A, Amaradio MD, Polosa R (2013). Electronic cigarette: a possible substitute for cigarette dependence. Monaldi Arch Chest Dis.

[23] Farsalinos KE, Romagna G, Tsiapras D, Kyrzopoulos S, Voudris V (2014). Characteristics, perceived side effects and benefits of electronic cigarette use: a worldwide survey of more than 19,000 consumers. Int J Environ Res Public Health.

[24] Farsalinos KE, Polosa R (2014). Safety evaluation and risk assessment of electronic cigarettes as tobacco cigarette substitutes: a systematic review. Ther Adv Drug Saf.

[25] Polosa R, Rodu B, Caponnetto P, Maglia M, Raciti C (2013). A fresh look at tobacco harm reduction: the case for the electronic cigarette. Harm Reduct J.

[26] Caponnetto P, Campagna D, Cibella F, Morjaria JB, Caruso M, Russo C, Polosa R (2013). EffiCiency and Safety of an eLectronic cigAreTte (ECLAT) as tobacco cigarettes substitute: a prospective 12-month randomized control design study. PLoS One.

[27] Polosa R, Morjaria JB, Caponnetto P, Campagna D, Russo C, Alamo A, Amaradio M, Fisichella A (2014). Effectiveness and tolerability of electronic cigarette in real-life: a 24-month prospective observational study. Intern Emerg Med.

[28] Bullen C, Howe C, Laugesen M, McRobbie H, Parag V, Williman J, Walker N (2013). Electronic cigarettes for smoking cessation: a randomised controlled trial. Lancet.

[29] Hartmann-Boyce J, McRobbie H, Bullen C, Begh R, Stead LF, Hajek P (2016). Electronic cigarettes for smoking cessation. Cochrane Database Syst Rev.

[30] Jones PW, Harding G, Berry P, Wiklund I, Chen WH, Kline Leidy N (2009). Development and first validation of the COPD Assessment Test. Eur Respir J.

[31] Kon SS, Canavan JL, Jones SE, Nolan CM, Clark AL, Dickson MJ, Haselden BM, Polkey MI, Man WD (2014). Minimum clinically important difference for the COPD Assessment Test: a prospective analysis. Lancet Respir Med.

[32] ATS statement: guidelines for the six-minute walk test. Am J Respir Crit Care Med. 2002;166(1):111–7. 10.1164/ajrccm.166.1.at1102 12091180

[33] Caponnetto P, Polosa R, Russo C, Leotta C, Campagna D (2011). Successful smoking cessation with electronic cigarettes in smokers with a documented history of recurring relapses: a case series. J Med Case Rep.

[34] Polosa R, Morjaria JB, Caponnetto P, Caruso M, Campagna D, Amaradio MD, Ciampi G, Russo C, Fisichella A (2016). Persisting long term benefits of smoking abstinence and reduction in asthmatic smokers who have switched to electronic cigarettes. Discov Med.

[35] Polosa R, Morjaria J, Caponnetto P, Caruso M, Strano S, Battaglia E, Russo C (2014). Effect of smoking abstinence and reduction in asthmatic smokers switching to electronic cigarettes: evidence for harm reversal. Int J Environ Res Public Health.

[36] Caponnetto P, Auditore R, Russo C, Cappello GC, Polosa R (2013). Impact of an electronic cigarette on smoking reduction and cessation in schizophrenic smokers: a prospective 12-month pilot study. Int J Environ Res Public Health.

[37] Godtfredsen NS, Lam TH, Hansel TT, Leon ME, Gray N, Dresler C, Burns DM, Prescott E, Vestbo J (2008). COPD-related morbidity and mortality after smoking cessation: status of the evidence. Eur Respir J.

[38] Godtfredsen NS, Vestbo J, Osler M, Prescott E (2002). Risk of hospital admission for COPD following smoking cessation and reduction: a Danish population study. Thorax.

[39] Au DH, Bryson CL, Chien JW, Sun H, Udris EM, Evans LE, Bradley KA (2009). The effects of smoking cessation on the risk of chronic obstructive pulmonary disease exacerbations. J Gen Intern Med.

[40] Anthonisen NR, Connett JE, Enright PL, Manfreda J (2002). Hospitalizations and mortality in the Lung Health Study. Am J Respir Crit Care Med.

[41] Kessler R, Faller M, Fourgaut G, Mennecier B, Weitzenblum E (1999). Predictive factors of hospitalization for acute exacerbation in a series of 64 patients with chronic obstructive pulmonary disease. Am J Respir Crit Care Med.

[42] Feldman C, Anderson R (2013). Cigarette smoking and mechanisms of susceptibility to infections of the respiratory tract and other organ systems. J Infect.

[43] Sopori M (2002). Effects of cigarette smoke on the immune system. Nat Rev Immunol.

[44] Wark PA, Johnston SL, Bucchieri F, Powell R, Puddicombe S, Laza-Stanca V, Holgate ST, Davies DE (2005). Asthmatic bronchial epithelial cells have a deficient innate immune response to infection with rhinovirus. J Exp Med.

[45] Campagna D, Amaradio MD, Sands MF, Polosa R. Respiratory infections and pneumonia: potential benefits of switching from smoking to vaping. Pneumonia. 2016;8(4). doi: 10.1186/s41479-016-0001-2. 10.1186/s41479-016-0001-2 PMC5469192 28702284

[46] Campagna D, Cibella F, Caponnetto P, Amaradio MD, Caruso M, Morjaria JB, Malerba M, Polosa R (2016). Changes in breathomics from a 1-year randomized smoking cessation trial of electronic cigarettes. Eur J Clin Investig.

[47] Scanlon PD, Connett JE, Waller LA, Altose MD, Bailey WC, Buist AS, Tashkin DP (2000). Smoking cessation and lung function in mild-to-moderate chronic obstructive pulmonary disease. The Lung Health Study. Am J Respir Crit Care Med.

[48] Tashkin DP, Rennard S, Taylor Hays J, Lawrence D, Marton JP, Lee TC (2011). Lung function and respiratory symptoms in a 1-year randomized smoking cessation trial of varenicline in COPD patients. Respir Med.

[49] Vestbo J, Edwards LD, Scanlon PD, Yates JC, Agusti A, Bakke P, Calverley PM, Celli B, Coxson HO, Crim C (2011). Changes in forced expiratory volume in 1 second over time in COPD. N Engl J Med.

[50] Greulich T, Koczulla AR, Nell C, Kehr K, Vogelmeier CF, Stojanovic D, Wittmann M, Schultz K (2015). Effect of a Three-Week Inpatient Rehabilitation Program on 544 Consecutive Patients with Very Severe COPD: A Retrospective Analysis. Respiration.

[51] Berkovitch A, Kivity S, Klempfner R, Segev S, Milwidsky A, Goldenberg I, Sidi Y, Maor E (2015). Time-dependent relation between smoking cessation and improved exercise tolerance in apparently healthy middle-age men and women. Eur J Prev Cardiol.

[52] Wong SL, Shields M, Leatherdale S, Malaison E, Hammond D (2012). Assessment of validity of self-reported smoking status. Health Rep.

